# The priority areas and possible pathways for health cooperation in BRICS countries

**DOI:** 10.1186/s41256-023-00318-x

**Published:** 2023-08-28

**Authors:** Zuokun Liu, Zongbin Wang, Ming Xu, Jiyan Ma, Yinuo Sun, Yangmu Huang

**Affiliations:** School of Public Health, Peking University, 38 Xue Yuan Road, Haidian District, Beijing, 100191 China

**Keywords:** BRICS countries, Health cooperation, Communicable disease, Access to medicine

## Abstract

As one of the largest alliances of middle-income countries, the BRICS, known as an acronym for five countries including “Brazil, Russia, India, China, and South Africa”, represents half of the global population. The health cooperation among BRICS countries will benefit their populations and other middle- and low-income countries. This study aims to summarize the current status of health cooperation in BRICS countries and identify opportunities to strengthen BRICS participation in global health governance. A literature review was conducted to analyze the status, progress, and challenges of BRICS' health cooperation. Content analysis was used to review the 2011–2021 annual joint declarations of the BRICS Health Ministers Meetings. The priority health areas were identified through segmental frequency analysis. Our research suggested that communicable diseases, access to medicine, and universal health coverage appeared most frequently in the content of declarations, indicating the possible top health priorities among BRICS' health collaboration. These priority areas align with the primary health challenges of each country, including the threats of double burden of diseases, as well as the need for improving health systems and access to medicines. Respective external cooperation, inter-BRICS health cooperation, and unified external cooperation are the main forms of health cooperation among BRICS countries. However, challenges such as the lack of a unified image and precise position, lack of practical impact, and weak discourse power have impeded the impact of BRICS on health governance. This study suggests that the BRICS countries should recognize their positioning, improve their unified image, and establish cooperative entities; at the same time, they should increase their practical strength, promote non-governmental cooperation, and expand the cooperation space through the “BRICS Plus” mechanism with countries with similar interests to join.

## Background

The concept of BRICS was first raised in 2001 as emerging economies, including Brazil, Russia, India, and China [1], and expanded to South Africa in 2010 [2]. The word “BRICS” is an acronym for the five countries. It is an essential club of middle-income countries with 42% of the global population and approximately 25% of the global economies. After years of rapid economic growth, the BRICS countries have made world-renowned achievements in health [3–5]. Their GDP growth has enabled BRICS countries to increase expenditures and investments in health, build infrastructure and improve life expectancy [6, 7].

As middle-income countries with large populations and geography, the BRICS countries share similar health development histories, challenges, and health aspirations. Domestically, BRICS countries are undergoing an unprecedented socioeconomic transformation and rapid urbanization [5]. The transformation has brought a shift in the spectrum of disease, from communicable diseases to non-communicable diseases [8]. The mortality rate and disease burden of non-communicable diseases in the BRICS countries have increased significantly in recent years [9, 10], while the threats of major communicable diseases and emerging infectious diseases (EIDs) are still significant challenges for the vulnerable populations, causing double burden of diseases in BRICS countries [11]. These similar public health challenges among the BRICS form the basis for their health cooperation [6]. As for foreign health cooperation, the BRICS countries have played an increasingly important role in regional and global health governance. All five countries are transforming from former aid recipient countries, and they all hope to demonstrate their strength through external cooperation, gaining a voice, and international recognition [12, 13]. Most of their foreign aid cooperation centered on neighboring countries, mainly focusing on technical cooperation, material and infrastructure assistance, and human resource training [14].

Although the BRICS countries share common needs for cooperation, the cooperation faces several challenges. From an economic perspective, their economic growth rate has gradually slowed compared to the golden decade from 2006 to 2016, which has harmed some of their health expenditures and the pharmaceutical market [15]. From a political point of view, some friction and confrontation within BRICS still exist [16]. The health cooperation among BRICS has mainly stagnated in commitment with limited clear policies or actions [17]. To cope with these challenges, the BRICS countries are constantly adjusting, deepening cooperation, and establishing mechanisms for technical cooperation in some health areas, such as COVID-19 and tuberculosis (TB) [18, 19].

Current health-related literature mainly focused on comparing the five countries' disease burden, health expenditure strategies, or health policy implementation. At the same time few studies have researched the BRICS cooperation and its impact on global health [6, 20, 21]. In addition, most studies only used the health indicators or policies of each country, and the review of BRICS cooperation documents was insufficient. The path of BRICS participation in global health governance remains unclear [11, 22, 23]. Thus, this study will focus on BRICS cooperation in health, explore the priority health areas, the motivation, the challenges and the possible cooperation pathways for BRICS countries on health.

## The priorities for BRICS cooperation in health areas

The BRICS countries have formally met annually to discuss health cooperation during the BRICS Health Ministers' meetings. A joint declaration would be issued by then as a guide for BRICS health cooperation and expressing common attitudes [24]. Therefore, all the 11 joint declarations, which were issued by the BRICS Health Ministers' meetings from 2011 to 2021, were studied using content analysis to review these documents. Frequency analysis of the paragraphs was used to identify the priority health areas. A total of 217 paragraphs were obtained, of which 158 were relevant to specific health areas.

**Fig. 1:**
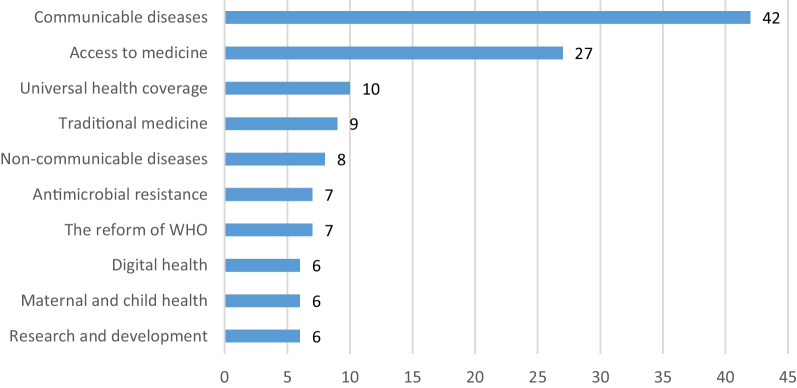
The total frequency of the top 10 concern areas appears in the joint Declaration

Figure 1 shows the top 10 health areas by frequency. “Communicable diseases (42 times)” and “Access to medicine (27 times)” were the two priority health areas with extremely prominent frequencies, while the others were roughly between 6 and 10 times with no significant difference. In the content of the BRICS declaration, “Communicable diseases” mainly included HIV/AIDS, TB, malaria, and EIDs such as Ebola and COVID-19 [25]. “Access to medicines” mainly involved enabling low-income and middle-income countries to obtain adequate and affordable medicines by promoting technology transfer, increasing the supply of medicines, and reducing prices [26].

BRICS countries have their considerations in selecting priority areas for cooperation. They set the health agendas in accordance with their characteristics and current challenges rather than simply following the West [20]. In fact, BRICS cooperation focused more on issues such as “Access to medicines” and “Traditional medicine,” which were not the main concerns of most high-income countries [20]. In general, the current priorities and focus of the BRICS health cooperation have the following characteristics: it is in line with the national conditions of the BRICS countries, the sustainable development goals, and the priorities of the WHO; meanwhile, it is in line with the concerns of the world's most populous low- and middle-income countries, and pays more attention to multilateralism and health equity.

## The reasons for BRICS health cooperation 

The above analysis shows that “Communicable diseases,” “Access to medicines,” and “Universal health coverage (UHC)” are currently the priorities of the BRICS countries. Although there are many other health issues equally important, the possible reasons for BRICS to emphasize these above areas might be because of the current common challenges and interests.

### The double burden of diseases

BRICS countries are all simultaneously facing the challenges of communicable and non-communicable diseases [11, 27]. Most BRICS countries have vast territories and cover several epidemic areas. Communicable diseases such as HIV/AIDS, TB, and EIDs still pose a serious threat and cause many deaths yearly [11]. BRICS countries accounted for three of the top ten countries with TB diagnoses in 2020, including India (2.59 million), China (0.84 million), and South Africa (0.33 million). Globally, HIV/AIDS is the only primary communicable disease that the prevalence rate of which is still increasing in BRICS countries [11]. South Africa has the worst HIV/AIDS epidemic, with the adult prevalence rate reaching 19.1% in 2020, resulting in a quarter of the country's deaths. Despite low adult prevalence rates, India (2.30 million), Russia (1.06 million), China (1.05 million), and Brazil (0.93 million) have large numbers of people living with HIV/AIDS [28]. The threat of EIDs cannot be ignored, such as SARS in China (2003) and Zika in Brazil (2015–2016). The COVID-19 pandemic in 2020 has further exposed the importance of combating EIDs [25, 29].

While eliminating communicable diseases, BRICS countries are also forced to face aging and non-communicable diseases as life expectancy increases and lifestyle changes [30, 31]. It can be extremely harmful to BRICS countries if non-communicable diseases are not controlled, as those in younger groups can negatively affect the workforce and economic development, while those in older groups pose a serious social burden [23]. Taking cancer as an example, 42% of cancer deaths worldwide occur in BRICS countries. Approximately $46.3 billion was lost in productivity a year, accounting for 0.33% of BRICS total GDP. The cancer problem in China is the most prominent among the five countries, with 23.7% of new cancer cases, and results in a significant loss of approximately $28.0 billion in productivity [32].

### Improving health systems

Immature health systems are mainly reflected in the inadequate coverage of primary health services and the unrealized universal health coverage [22]. For achieving UHC, the health system is inadequate in the five countries and has room for improvement. China has made remarkable achievements in universal health coverage but is facing a shift in the spectrum of diseases and aging problem [3, 33]. Russia has established a health financing mechanism with free medical care, but the out-of-pocket of ordinary people has increased in recent years [7, 22]. Brazil faces huge disparities in public and private health insurance, thereby funding for health spending is also becoming a problem [34]. The health systems of South Africa and India are more vulnerable and unable to meet the basic health needs of their populations [3, 7]. BRICS countries are undergoing rapid urbanization, and a complete primary health system is necessary to ensure the health of citizens. However, achieving universal health coverage is extremely difficult due to the large population base and complex social structure [3]. At present, the BRICS countries are willing to take the realization of universal health coverage as one of the most important goals and exchange experiences especially on health system strengthening [35].

### Access to medicines

Despite the fact that BRICS countries have the world's largest emerging pharmaceutical markets and increasing consumption levels, they are still in a passive position in the global pharmaceutical industry. At the pharmaceutical Research and Development (R&D) stage, the capacity for innovative drug research is challenging to be improved in the short term due to the lack of accumulation of technology and talents [36, 37]. Brazil’s lack of initiative in researching and producing vaccines during the COVID-19 pandemic was a wake-up call [38]. Therefore, the independent development of original medical products is a shared vision among BRICS. The BRICS countries are at the core of the world in the pharmaceutical manufacturing stage, and the pharmaceutical industry organizations of various countries are complementary [39, 40]. Take China and India as an example, the exportation of active pharmaceutical ingredients (APIs) from China accounts for 30% of the global value. Meanwhile, India is complicated in producing APIs locally for geographical location and climate conditions, resulting in 70% of APIs importation relying on China [41]. However, Indian pharmaceutical enterprises are more active in the global market than China. From 2009 to 2019, the Food and Drug Administration (FDA) approved 2216 abbreviated new drug applications (ANDA) in India, accounting for 34% of the total [42, 43]. Traditional medicine is also an essential direction of cooperation. BRICS countries have a foundation for traditional medicine markets and have developed rapidly in patent applications and industrial supply. Cooperation in developing traditional medicine and registered herbal medicine patents are important ways to gain the initiative for the BRICS [44].

## Modalities for BRICS health cooperation

BRICS cooperations mainly include three pathways. In addition to their respective external cooperation, internal and unified external cooperation are the main pathways to participate in global health governance under the BRICS framework. Among them, Internal cooperation mainly refers to exchanges or organization building among BRICS countries, while unified external cooperation mainly refers to the interaction between BRICS countries and other actors. In addition, BRICS countries cooperated in issuing political commitments and facilitating international expert working groups, etc.

### Respective external cooperation

The five countries were implementing respective external cooperation with different priority collaborating regions and areas. For example, China has started health development cooperation with Africa, Asia, and Latin America since the 1960s by dispatching Chinese medical teams (CMTs) and has now expanded to building medical facilities, training medical personnel, providing medical products, et al. [45]. Russia has mainly provided bilateral development assistance to the Commonwealth of Independent States. The core of its health assistance is communicable diseases, and the main forms of cooperation are strengthening laboratory capacity, vaccine and diagnostic reagent development, personnel training, and technical cooperation [46]. India has mainly provided health aid to Southeast Asia and Central Asia and has sought to partner with Africa through the India-Africa Forum Summit (IAFS) in recent years. India mainly provides financial aid and credit, medical supplies, and technical cooperation for health in other middle- and low-income countries [17]. Brazil’s foreign health cooperation mainly supports South American countries by improving technical cooperation in healthcare, enhancing access to free medication and vaccines, and promoting the Human Milk Banks [47]. South Africa mainly cooperates with African countries in health, especially in Sub-Saharan Africa. The theme of South Africa's health cooperation mainly focuses on HIV/AIDS and TB. The primary forms are technical cooperation, laboratory capacity building, and health workforce training [48].

### Inter- BRICS health cooperation

Internal cooperation mainly refers to building technical cooperation platforms, developing pharmaceutical products, sharing epidemic data, and exchanging academic experience. In the declaration, BRICS planned to create a drug research and development alliance for TB, HIV/AIDS, and malaria, aiming to obtain proprietary, innovative drugs to control these diseases. For example, ‘The BRICS TB Research Network” is a platform for collaboration, particularly in clinical treatment and capacity building, through the systematic use of existing expertise and indigenous technology within the BRICS countries [18]. Moreover, the BRICS countries plan to launch a communicable disease surveillance system to share epidemic data and information to prevent pandemics and control outbreaks in a better way. BRICS built the BRICS Vaccine Research Centre in 2021 and committed to delivering vaccines as a global public good and increasing equity in access [49]. The BRICS countries also established the BRICS Committee for International Cooperation in Health (BCICH), which aims to promote academic exchanges in the field of traditional medicine among medical personnel from BRICS countries [50].

### Unified external cooperation

Unified external cooperation mainly refers to cooperating with the WHO and other international health organizations and stating attitudes through joint declaration. BRICS countries support the leading and coordinating role of the WHO in global health governance. The delegations of BRICS countries have organized the BRICS meeting during the World Health Assembly (WHA) to strengthen links with the WHO [51]. BRICS countries were actively exploring opportunities to cooperate with the WHO in multiple areas, including tobacco control, technology transfer, and communicable disease surveillance systems [4]. The BRICS also actively supported the reform of the WHO and stood with other middle- and low-income countries. In addition, BRICS was more than a solid political union, so soft power tends to be used more than direct policy influence [20]. Expressing attitude on a health topic by releasing joint statements was the main way for BRICS countries to expand their international influence. They also reaffirmed the previous joint declarations and acknowledged significant global health achievements in that year to raise global awareness of health-related issues [21].

## Challenges for BRICS health cooperation

Our analysis suggested that BRICS countries have already carried out cooperation in various priority health areas, including communicable diseases and access to medicines, and have recognized the motivation and paths of cooperation. Based on that, we hope to analyze the current challenges of the BRICS for better cooperation in health.

The overall image of BRICS cooperation is not significant because they participated in global health governance mainly as different individual countries [17]. At present, except for the two scheduled meetings, BRICS Health Ministers Meeting and side meeting on WHA, the BRICS countries rarely present as a whole group in international statements and actions. As a result, the BRICS countries are generally seen as five separate countries rather than an entirety. The lack of a monolithic image of BRICS stems from the unclear, common roles. The BRICS were once seen as emerging markets. However, they have lost the original name of “high-speed growth economy” to some extent [52, 53]. Thus, re-understanding their strengths and position is an important task, and undertaking more unified action to show their roles.

BRICS cooperation mostly remains at the level of meetings and declarations, contrary to ambitious plans in the joint declaration. At present, BRICS cooperation still needs more specific responsibility bodies, effective financing mechanisms, and regulatory measures, to carry out practical health activities. BRICS countries currently like to use words such as “recognize” and “emphasize” to express their shared vision, but vision without sustainable action is not enough [4, 6]. It is often difficult for most international organizations to achieve practical results. Research showed that the G7 and G20 seemed to have the same challenge [20]. Currently, after the COVID-19 pandemic, BRICS countries have already started action since 2020, such as an early warning system for diseases and a vaccine research and development center [19]. However, whether these actions are planned long-term collaborations or short-term responses to the pandemic remains unknown.

BRICS countries have vast populations and economies, but their discourse power does not match it accordingly. In fact, in the health area, the international performance of BRICS countries is not active. In BRICS cooperation affairs, the proportion of internal cooperation generally exceeds that of external cooperation [20]. Meanwhile, external cooperation still focuses on dialog and expressing attitude. The problem has to do with the loosely organized nature of the BRICS [54]. However, all five countries have a foundation for external cooperation and a desire to exert more international influence. BRICS should play a more critical role in global health governance as a whole to speak up for developing countries.

## Possible pathways for BRICS future health cooperation

Based on the current complex international situation, some possible suggestions were put forward to establish a more effective BRICS health cooperation.

### Clearing position and establishing a unified international image

Recognizing and understanding the BRICS countries' current position in global health governance is a prerequisite for enhancing the unified image. BRICS cooperation in health reflects not only the emerging development of the BRICS countries themselves, but also as a platform that carries the expectations of other emerging or middle- and low-income countries. Therefore, these countries' common position and collective will should be better reflected through BRICS cooperation [12, 55]. However, even if the positioning is precise, some concrete measures are needed to project the image, not as individual countries but as BRICS. BRICS countries should strengthen their internal trust and make a statement with a more unified voice in the international arena, such as the WHA and United Nations (UN) General Assembly High-Level Meeting on the fight against TB, et al [20]. Delegates can support each other in speeches to demonstrate a common attitude of BRICS. In addition, advocacy efforts also need to be highlighted to inform the outside world about the outcomes of BRICS health cooperation.

### Deepening existing cooperation and expanding scope

BRICS health cooperation should build a more flexible dialog mechanism. Meetings twice a year are still insufficient to meet the ambitious cooperation plans proposed in priority areas. Simultaneously, it is recommended that BRICS countries carry out systematic organization building and implement action plans with specific national responsibilities to improve operational efficiency. In addition, a performance evaluation system and funding mechanisms should also be established to ensure substantial practical outcomes. Promoting links between the private sector is also a step to deepen cooperation. BRICS cooperation should create more opportunities for the private sector to communicate actively through platforms. Relevant medical enterprises in pharmaceutical, digital medical, and human resources training can all participate. In addition, enterprises, philanthropies, and other non-government organizations of BRICS countries can also become long-term actors and funding providers to enhance practical cooperation. Establishing research funds is also viable to meet the common scientific research needs, especially for middle- and low-income countries [56].

In addition to deepening cooperation in current priority areas, broadening new areas of focus should be attached to great importance. With further socio-economic development and the transformation of the spectrum of the disease, BRICS cooperation should consider adding other health areas of focus in the near future. Studies have shown that the ecological footprint of BRICS countries is decreasing, which means that green health may become a new priority [57]. There is also growing concern about mental health in BRICS countries, which has been exacerbated by the COVID-19 pandemic [58]. Furthermore, with more people moving into the cities, the health challenges brought by urbanization should also be noticed.

### Strengthen external cooperation through the “BRICS Plus”

The “BRICS Plus” mechanism means that BRICS will further strengthen liaison, interaction, dialog, and cooperation with other middle-income countries and is a powerful mechanism to broaden the space for external cooperation. On the one hand, “BRICS plus” makes it possible for BRICS to cooperate with other political entities as a new relationship of partnership and non-alliance beyond the old formula of a political and military alliance. On the other hand, “BRICS plus” will also increase BRICS's strength and expand its discourse power and international influence. During the Xiamen Summit in 2017, when the “BRICS Plus” proposal was brought up, leaders of Mexico, Egypt, Guinea, Thailand, and Tajikistan were first invited to participate in the BRICS meeting [59, 60]. It has increasingly become a consensus that the BRICS shall incorporate more-representative middle- and low-income countries. These representative countries could implement a positive impact on the surrounding area through regional radiation. Including representative countries from each region in BRICS cooperation will bring the world closer and better coordinate their responses to global health threats. By coordination, BRICS cooperation can serve as a superior overarching mechanism and as a platform for south-south health cooperation and consultation in various regions.

## Limitations

Due to the limited availability of the documents, this paper may overlook some collaboration projects, especially those not been publicly reported, thus underestimating the extent of actual cooperation. In addition, this study mainly focuses on health, but BRICS cooperation is actually a complex issue covering many fields. Therefore, health cooperation can only represent genuine BRICS cooperation in part of the field. It is hoped that future research could put forward health strategies and recommendations for BRICS countries in a higher strategic context.

## Conclusions

This study focused on the status quo, challenges, and development paths of BRICS countries in health cooperation. BRICS countries have participated in global health governance activities in various priority health areas. However, the current cooperation still faces challenges such as lack of clear overall image and actual action. The BRICS should focus on building an overall image, establishing practical and effective cooperation, and increasing communication channels among private enterprises. Meanwhile, expanding membership and cooperation through “BRICS plus” is a possible pathway to improve the health of a larger population and contribute more to global health governance.

## Data Availability

Not applicable.

## Abbreviations

BRICS: Brazil, Russian, India, China and South Africa
TB: Tuberculosis
HIV/AIDS: Human Immunodeficiency Virus/Acquired Immune Deficiency Syndrome
WHO: World Health Organization
WHA: World Health Assembly
R&D: Research and Development
UN: United Nations
FDA: Food and Drug Administration (America)
CMTs: Chinese Medical Teams
EIDS: Emerging infectious diseases
APIs: Active pharmaceutical ingredients
COVID-19: Coronavirus disease 2019
ANDA: Abbreviated New Drug Application
